# ﻿A new *Trychopeplus* species (Phasmatodea, Diapheromerinae, Cladomorformia) discovered from Ecuador’s enigmatic Chocó ecoregion

**DOI:** 10.3897/zookeys.1217.130397

**Published:** 2024-11-12

**Authors:** Oskar V. Conle, Pablo Valero, Frank H. Hennemann

**Affiliations:** 1 The Bavarian State Collection of Zoology, Munich, Germany The Bavarian State Collection of Zoology Munich Germany; 2 University of Murcia, Murcia, Spain University of Murcia Murcia Spain; 3 Zoologische Staatssammlung München, Munchen, Germany Zoologische Staatssammlung München Munchen Germany

**Keywords:** Identification key, Insecta, stick insects, taxonomy, morphology

## Abstract

This study presents a description of a new stick insect species belonging to the genus *Trychopeplus* Shelford, 1909, discovered by the authors in the Chocó ecoregion of northwestern Ecuador. *Trychopeplusmashpiensis***sp. nov.** is described and illustrated based on males, females, and eggs. The distinctive features of this new species, such as its unique body ornamentation and the morphology of its egg structure without fringes, clearly differentiate it from other known species within the genus. Photographs of the new species are provided, along with an updated key and distribution map for all *Trychopeplus* species. These findings enhance our understanding of the genus’s diversity.

## ﻿Introduction

*Trychopeplus* Shelford, 1909, is a genus of Neotropical stick insects (order Phasmida Leach, 1815) known for its remarkable morphological adaptations that allow it to blend almost perfectly with the epiphytic mosses in its habitat. These adaptations make *Trychopeplus* one of the most cryptic stick insect genera (Hennemann and Conle 2024).

The genus was described by Shelford in 1909 to distinguish Neotropical species from *Pericentrus* Redtenbacher, 1908, based on morphological differences and distinct geographic distributions compared to the type species *Pericentrusmoewisi* Redtenbacher, 1908. The known distribution of *Pericentrus* is confined to East and South Asia, while *Trychopeplus* is endemic to the Neotropics (Zompro 2001; Brock and Büscher 2022; Hennemann and Conle 2024).

Traditional taxonomic revisions by Zompro (1998) and more recent work by Hennemann and Conle (2024) have confirmed the classification of *Trychopeplus* within the subfamily Diapheromerinae Kirby, 1904. Additionally, molecular phylogenetic studies, such as those by Bank and Bradler (2022) and Forni et al. (2022), have supported this classification, positioning *Trychopeplus* as closely related to *Phanocles* Stål, 1875. Despite this phylogenetic relationship, *Trychopeplus* is morphologically distinct due to several specialisations, including irregularly foliaceous lobes, spines, and teeth on the head, body, and limbs, adaptations that enhance its ability to camouflage effectively in its natural habitat of moist, mossy forests (Hennemann and Conle 2024).

Until now, the only eggs described from the genus were those of the type species *Trychopepluslaciniatus* (Westwood, 1874), which were characterised by being entirely covered with long fringes, a trait previously considered a potential autapomorphy of the genus (Hennemann and Conle 2024).

This study aims to describe a new species, *Trychopeplusmashpiensis* sp. nov., based on specimens collected from the Chocó forests in northwestern Ecuador. Through a comprehensive morphological analysis of males, females, and eggs, distinctive features are described and illustrated, which not only distinguish this new species from its congeners but also provide crucial insights into intrageneric variability. Furthermore, an updated key to all known *Trychopeplus* species is presented, along with a distribution map for the genus.

## ﻿Material and methods

The material used in this study was collected through nocturnal direct sampling on trails near Mashpi Lodge, located in the parish of Pacto, northwest of the Ecuadorian province of Pichincha. Samplings took place from 1–4 October, 2023, under the collection permit MAATE–ARSFC–2023–3348.

The adult females collected were kept in a terrarium for several days to lay eggs. Euthanasia was performed by immersing the specimens in 96% ethanol for a few minutes. Both adults and eggs were stored dry, with adult specimens pinned. Insects and eggs were examined using a stereoscope (Zeiss Stemi SV6). Measurements were taken with a digital caliper at a precision of 0.1 mm. All eggs examined were fully developed and already laid. The terminology used for the descriptions of egg structures follows that of Clark-Sellick (1997, 1998). The terminology for the description of the genital morphology follows Bradler (2009). Photographs of live and dry specimens were taken with a Sony A7RIII camera fitted with a Tamron 90 mm f/2.8 DI VC USD macro lens. Lighting was provided by a Godox V350 flash with a diffuser. In photographs with a white background, backlighting was provided by a 24W 6000K LED light panel. Sample depositories and type status are abbreviated as follows:

**ZSFQ**Museo de Zoología de la Universidad San Francisco de Quito, Quito, Ecuador;

**ZSM**Zoologische Staatssammlung München, Munich, Germany.

## ﻿Results

**Diapheromeridae**

**Diapheromerinae Kirby, 1904**

**Cladomorformia**

### ﻿ Trychopeplus

Taxon classificationAnimaliaPhasmatodeaDiapheromeridae

Shelford, 1909

9298AC0A-29EE-5757-BA0B-1752D1AB0DFB

#### Type species.

*Pericentrusmultilobatus* Redtenbacher, 1908: 352 (= *Ceroyslaciniatus* Westwood, 1874: 174), by original monotypy.

*Trychopeplus* Brunner v. Wattenwyl, in litt. Shelford 1909: 354, 356 & pl. 6: 5 (♂). Hebard 1922: 358, pl. 15: 5 & 6 (♀ & egg). Hebard 1924: 148, pl. 6: 8 & 9 (♀). Campos 1926: 15 & plate (♀). Zompro 2001: 230. Otte and Brock 2005: 338. Conle, Hennemann and Gutiérrez 2011: 56. Brock and Büscher 2022: 515. Hennemann and Conle 2024: 283.

*Ceroys* Westwood, 1874: 174, pl. 32: 4 (♀).

*Parobrimus* Kirby, 1904: 344.

*Pericentrus* Redtenbacher, 1908: 352 (in part). Shelford, 1909: 356.

*Trichopeplus*Bradley and Galil 1977: 180 (misspelling).

#### Species included.

*Trychopepluslaciniatus* (Westwood, 1874: 174, pl. 32: 4) [Nicaragua, Costa Rica, Panama]

= *Pericentrusmultilobatus* Redtenbacher, 1908: 352

*Trychopeplusspinosolobatus* (Redtenbacher, 1908: 353) [Venezuela]

= *Pericentrusappendiculatus* Redtenbacher, 1908: 353.

*Trychopeplusthaumasius* Hebard, 1924: 148, pl. 6: 8 & 9 [Colombia, Ecuador]

= *Trychopeplusortho-lamellatus* Campos, 1926: 15 & pl.

*Trychopeplusmashpiensis* Conle, Valero & Hennemann sp. nov. [Ecuador]

### ﻿ Trychopeplus mashpiensis

Taxon classificationAnimaliaPhasmatodeaDiapheromeridae

Conle, Valero & Hennemann sp. nov.

4554DDCA-B2D8-52EB-AEBA-0B408072F225

https://zoobank.org/F2317335-BBE0-4CBF-8A42-3363832F8965

Figs 2 , 3 , 4 , 5 , 6 , 7

#### Material examined.

***Holotype*** • ♂; Ecuador: Prov. Pichincha, Parroquia de Pacto, Mashpi Lodge; 0°09'58.0"N, 78°52'38.4"W; 800–1000 m; leg. Cisneros, Conle, Valero & Hennemann 1–4.10.2023. [ZSFQ]. ***Paratype*** • 1 ♂, 3 ♀♀; 10 eggs: Ecuador: Prov. Pichincha, Parroquia de Pacto, Mashpi Lodge; 0°09'58.0"N, 78°52'38.4"W; 800–1000 m; leg. Cisneros, Conle, Valero & Hennemann 1–4.10.2023. [ZSFQ]. ***Paratype*** • 2 ♂♂, 2 ♀♀; 10 eggs: Ecuador: Prov. Pichincha, Parroquia de Pacto, Mashpi Lodge; 0°09'58.0"N, 78°52'38.4"W; 800–1000 m; leg. Cisneros, Conle, Valero & Hennemann 1–4.10.2023. [ZSM].

#### Differential diagnosis.

**Figure 1.**
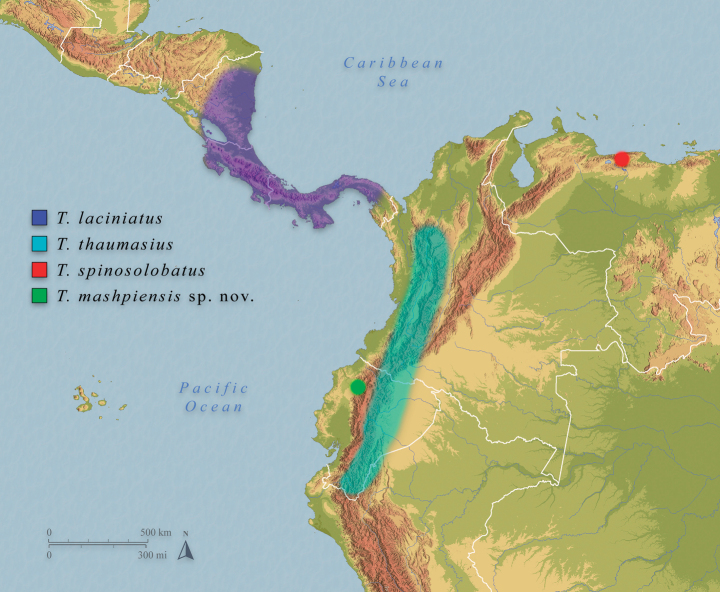
Approximate known distribution of four species of *Trychopeplus* Shelford, 1909 based on available literature.

**Figure 2.**
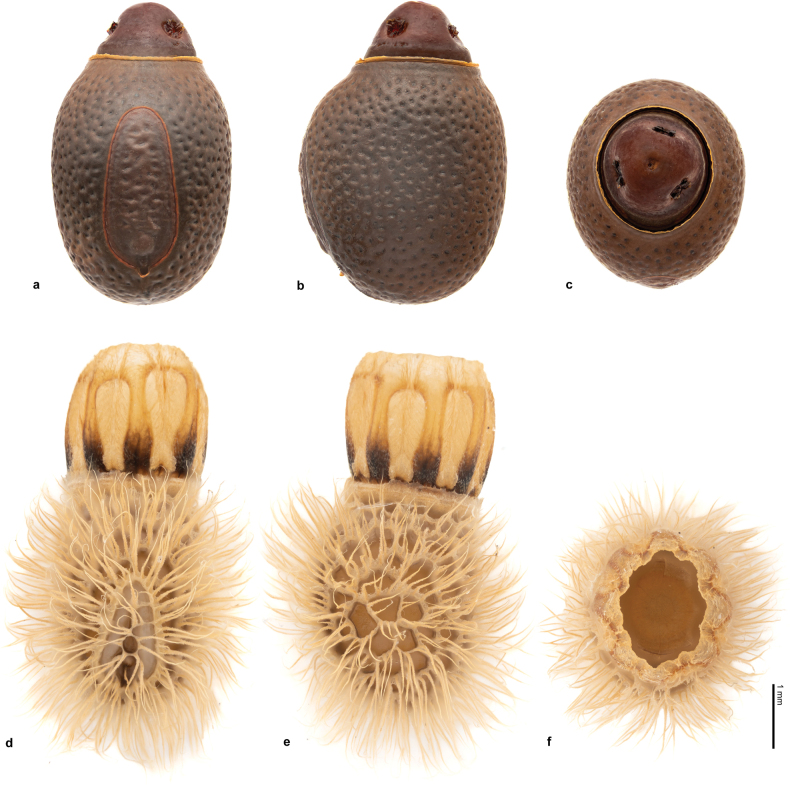
Egg of *Trychopeplusmashpiensis* sp. nov. in dorsal (**a**), lateral (**b**) and anterior (**c**) view. Egg of *Trychopepluslaciniatus* (Westwood, 1874) in dorsal (**d**), lateral (**e**) and anterior (**f**) view.

*T.mashpiensis* sp. nov. is clearly distinguishable from *T.laciniatus* and *T.thaumasius* by the shape of its ornamentation, which is spiny in the new species and consists of irregular foliar lobes in *T.laciniatus* and *T.thaumasius*. Additionally, the distribution of *T.laciniatus* appears to be restricted to Central America (Fig. 1). Furthermore, the eggs of *T.laciniatus* and *T.thaumasius* have capsules densely covered with long, hair-like yellowish fringes, whereas in *T.mashpiensis* sp. nov. the capsule surface is smooth (Fig. 2).

**Figure 3.**
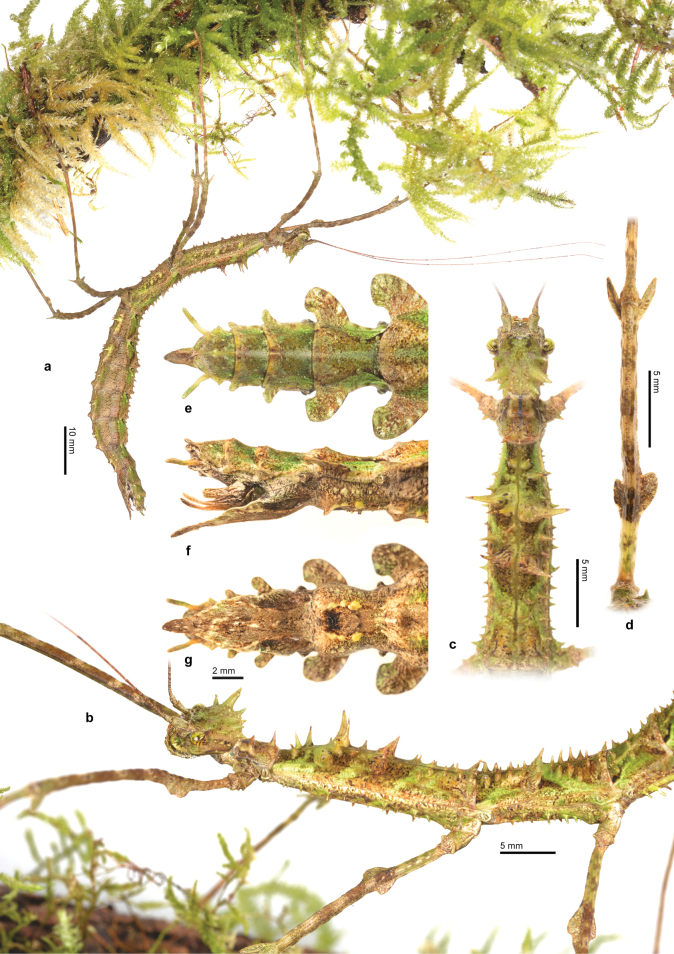
Female of *Trychopeplusmashpiensis* sp. nov. **a** habitus in lateral view **b** head and thorax in dorsolateral view **c** head, pro- and mesothorax in dorsal view **d** mesofemora in dorsal view **e** end of the abdomen in dorsal view **f** end of the abdomen in lateral view **g** end of the abdomen in ventral view.

**Figure 4.**
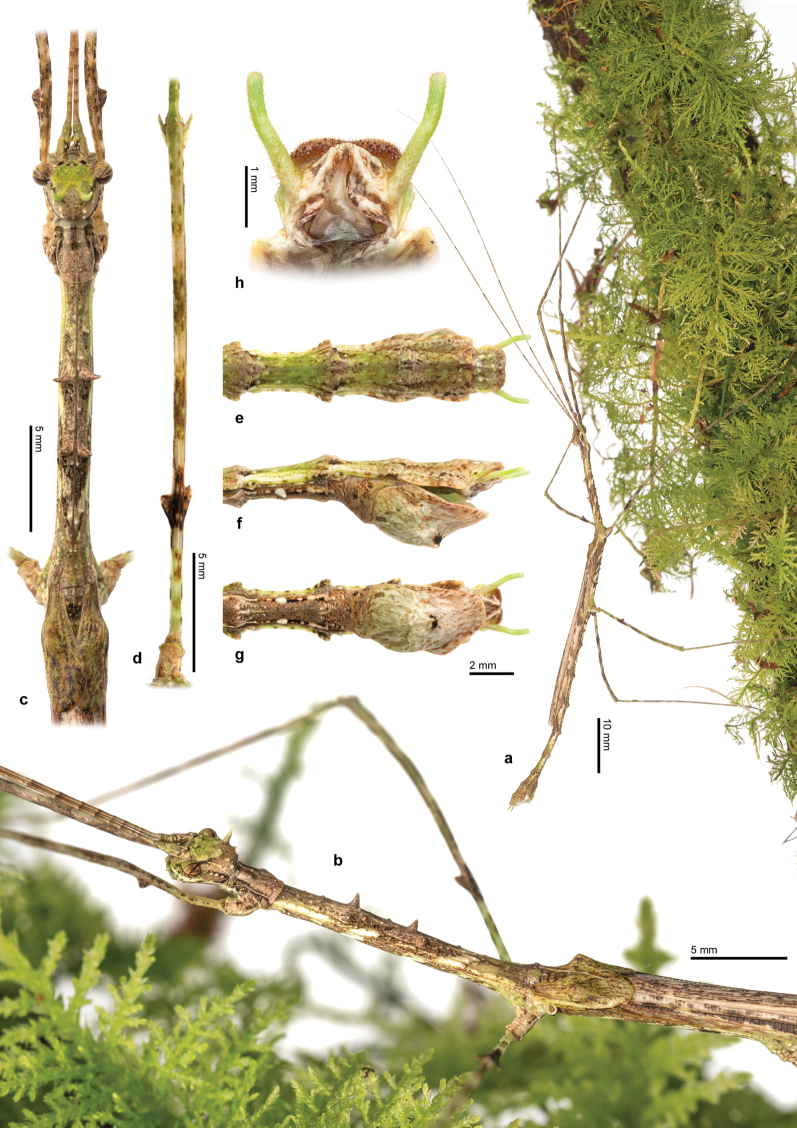
Male of *Trychopeplusmashpiensis* sp. nov. **a** habitus in lateral view **b** head and thorax in dorsolateral view **c** head, pro- and mesothorax in dorsal view **d** mesofemora in dorsal view **e** end of the abdomen in dorsal view **f** end of the abdomen in lateral view **g** end of the abdomen in ventral view **h** detail of the vomer.

The female of the new species is similar to *T.spinosolobatus*, of which the male and egg are unknown. However, they can be easily differentiated by the shape of the spines, the ornamentation of the femora, and the length of the subgenital plate. In *T.mashpiensis* sp. nov., the tips of the body spines are acute, while in *T.spinosolobatus* they are clearly rounded. The femoral ornamentation of *T.mashpiensis* sp. nov. consists only of two pairs of foliar lobes (Figs 3D, 4D), whereas *T.spinosolobatus* has four, which are also larger. As for the subgenital plate, in *T.mashpiensis* sp. nov. it is approximately 1.2 times the length of the last three tergites combined, whereas in *T.spinosolobatus* it is much longer, measuring about 1.7 times the length of the last three tergites. Additionally, the distribution of *T.spinosolobatus*, although somewhat uncertain, seems to encompass Venezuela and Colombia (Hennemann and Conle 2024), while the new species is only known from the northwest of Ecuador (Fig. 1).

#### Description.

The colouration is described mostly from photographs of live specimens. Table 1 presents the detailed measurements of the Holotype, as well as the measurement ranges of the Paratypes deposited in two official collections.

**Table 1.** Measurements (mm) of *Trychopeplusmashpiensis* sp. nov. HT = holotype; PT = paratype.

	♂, HT [ZSFQ]	♂♂, PT [ZSFQ]	♀♀, PT [ZSFQ]	♂♂, PT [ZSM]	♀♀, PT [ZSM]
Body (incl. subgen. pl.):	–	–	81.5–87.4	–	83.6–85.7
Body:	73.3	72.3–74.3	79.6–85.6	72.6–73.9	82.1–83.0
Pronotum:	2.5	2.7–3.1	3.7–4.5	2.7–2.8	4.1–4.3
Mesonotum:	14.1	14.5–14.7	18.0–19.4	14.1–14.3	17.7–19.0
Metanotum:	4.1	3.6–4.3	5.6–6.2	3.0–4.4	6.2–7.4
Median segment:	10.5	10.7–10.9	6.4–7.1	9.4–10.3	4.4–6.4
Tegmina:	5.8	6.3–6.5	–	5.6–5.7	–
Alae:	35.7	34.5–37.7	–	34.9–36.8	–
Profemora:	27.6	27.2–28.0	23.9–26.2	26.4–28.2	24.6–26.3
Mesofemora:	21.3	22.1–22.4	17.8–18.8	22.3–23.0	19.1–20.6
Metafemora:	25.2	26.1–26.9	22.1–22.3	26.5–27.6	23.3–24.5
Protibiae:	30.5	30.9–31.0	25.4–28.0	30.6–31.1	26.1–29.2
Mesotibiae:	22.3	22.6–22.9	19.7–21.3	22.6–22.8	19.9–23.1
Metatibiae:	27.8	28.3–28.9	26.0–28.8	28.9–29.0	26.3–32.0
Antennae:	74.1	74.5–75.2	23.8–61.1	74.2–75.3	62.1–70.6

**Figure 5.**
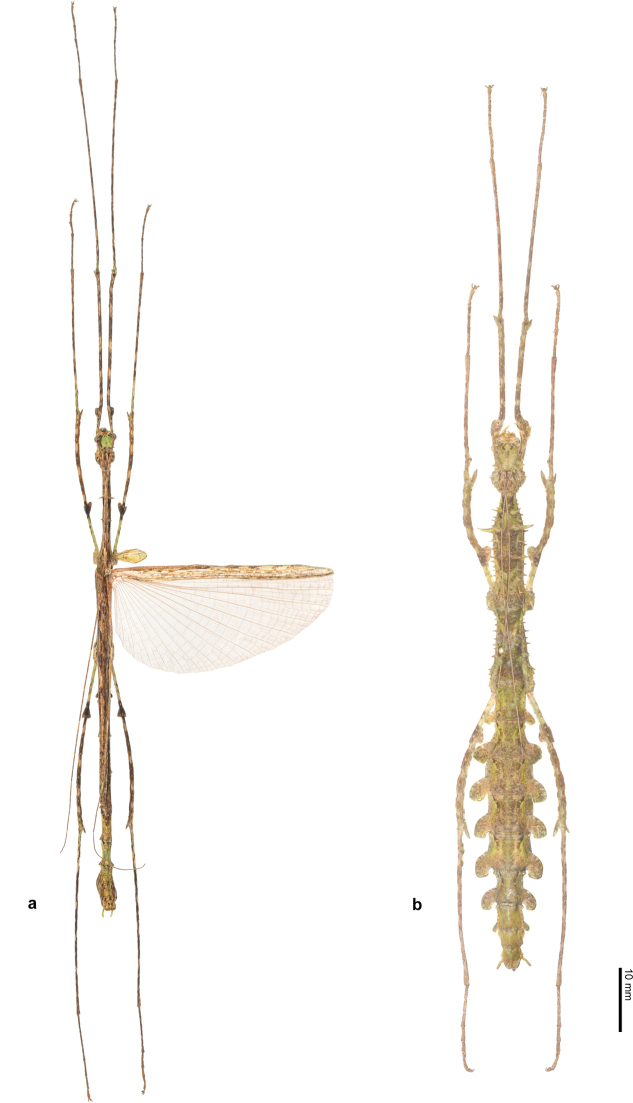
Habitus of *Trychopeplusmashpiensis* sp. nov. in dorsal view **a** male holotype **b** female paratype.

**Figure 6.**
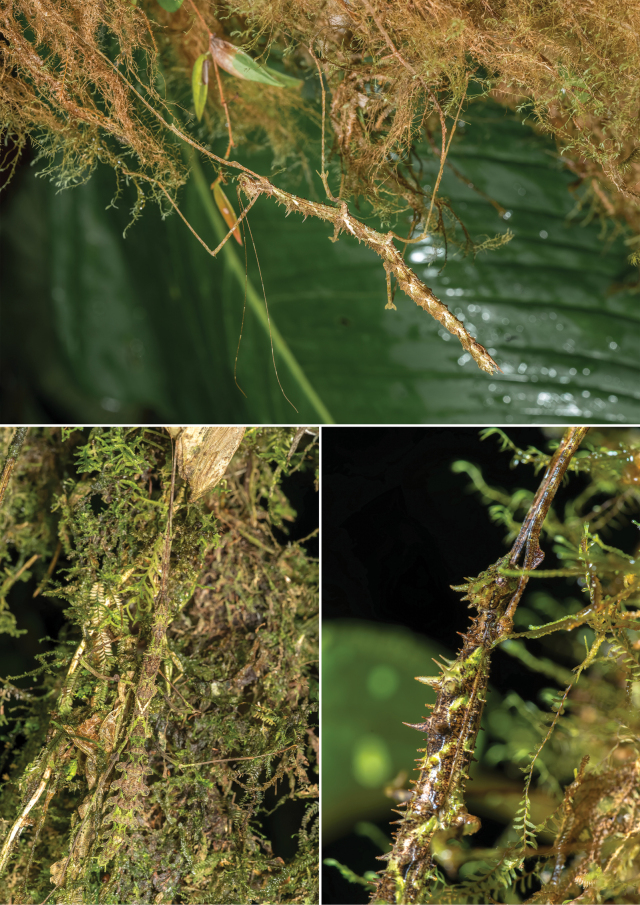
Females of *Trychopeplusmashpiensis* sp. nov. in their natural habitat.

♀♀ (Figs 3, 5, 6). Of medium size for the genus (body length 79.6–85.6 mm), it features numerous spines of varying sizes across the head, thorax, and abdomen; and a relatively short subgenital plate for the genus. Its overall body and limb colouration range from medium to dark brown and olive green, with an irregular pattern occasionally speckled with small white or cream marks. Lime green eyes exhibit irregular dark brown lines. Antennae can be divided into a pattern formed out of groups of antennomeres, gradually darkening towards the apex of each group of antennomeres. The pronotum displays a thin, black, dorsolongitudinal line that gradually fades or even disappears towards its posterior half. In dorsal view, the metanotum displays two cream-coloured diagonal bands arranged in a “V” shape, with the apex touching the posterior margin of the metanotum.

***Head*.** The head is ovoid, with the occiput strongly convex, approximately 1.4 times longer than wide. The vertex features two dorsolateral formations of spines or conical protuberances, arranged more or less in line, two of which are prominently larger and slightly pointing towards the posterior. Additionally, irregular and much smaller conical protuberances are present between these formations, as well as on the genae. The eyes are medium-sized, nearly circular in shape, projecting almost hemispherically, and their diameter is approximately twice the length of the genae. The antennae are filiform and reach the posterior margin of abdominal segment V. The scape is dorsoventrally compressed, with the outer lateral margin gently concave and approximately 2.1 times longer than wide. The pedicel is round in cross-section and somewhat constricted apically.

***Thorax*.** The entire thorax is densely covered with spine-like protuberances, varying in size and generally exhibiting a degree of bilateral symmetry. The pronotum is approximately 0.75 times the length of the head and 0.9 times its width, with a basically rectangular shape and a slight premedial narrowing. The anterior margin is somewhat elevated. The transverse median sulcus is moderately distinct, curved, and nearly expanding across the entire width of the segment. It features several dorsal spines, arranged approximately parallel to each other; the first pair of spines are simple and small, located just before the transverse median sulcus; the second pair is situated near the posterior margin of the pronotum, larger in size and appearing paired, almost as if forming a single bifurcated structure. The mesothorax is approximately 4.3 times longer than the prothorax and of uniform diameter except for a slight anterior narrowing. Among the spines of the mesonotum, the most prominent are located approximately at the distance of the first and second quarters of its total length. The metanotum is approximately one-third the length of the mesonotum. The posterior margins of the meso- and metanotum exhibit two dorsal spines each, parallel-sided and of medium size, slightly directed backwards.

***Abdomen*.** The abdomen (excluding the subgenital plate) is approximately the same length as the combination of the head and thorax (including the median segment). The median segment is roughly the same size as the metanotum and is more or less quadrangular. Segments II–IX feature prominent dorsoventrally flattened lobes, with rounded margins on the posterior sides of each segment. These lobes gradually increase in size from segment II to VI, and then gradually decrease until segment IX. Segment II is slightly shorter than the median segment and slightly longer than wide. Segments II–V have approximately the same length and width, while from VI to IX, they gradually decrease in length and width. Sternites II–VII exhibit small blunt spines and a medial protuberance on the posterior margin of each segment, covered by small setae. The preopercular organ is barely distinguishable and consists simply of a small, wart-like swelling near the posterior margin of sternite VII; the entire area is dark reddish-brown. The anal segment is slightly longer than tergum IX and has a generally rounded outline, except for a small posterior medial incision and two triangular lateral projections near the base. The epiproct is very small and almost completely hidden beneath the anal segment. The cerci are of similar length to the anal segment, thin, and circular in cross-section; extending clearly beyond the posterior margin of the anal segment. The gonapophyses are moderately enlarged, keeled, and almost reaching the base of the cerci. Gonapophysis VIII is relatively long and slightly curved upwards, reaching approximately the posterior margin of the anal segment or even slightly surpassing it, but never reaching the apex of the subgenital plate. The subgenital plate is shovel-shaped, slightly longitudinally keeled, and projecting approximately 20% of its total length over the apex of the anal segment; the lateral margins are undulated and the apex is slightly curved downwards.

***Legs*.** All legs are long and slender. Both femora and tibiae are trapezoidal in cross-section, with the two dorsal carinae closely spaced. All femora are strongly curved. Additionally, the profemora are also strongly curved at the base to accommodate the head when aligned in line with the body. The meso- and metafemora feature two pairs of foliaceous lobes on the ventral carinae, one on the outer ventral carina and another on the inner, with rounded edges. The first pair is located approximately at one-third from the base, consisting of semicircular lobes. The second pair is positioned at the posterior end of the profemora, and they are at least twice as long as they are wide. In the profemora, the lobes are similarly arranged but only present on the outer ventral carina. All femora are longer than the mesothorax. All tibiae are slender, more or less straight, slightly longer than the corresponding femur, and lack ornamentation. The basitarsi are slightly shorter than the combined remaining tarsomeres and also lack ornamentation.

**Figure 7.**
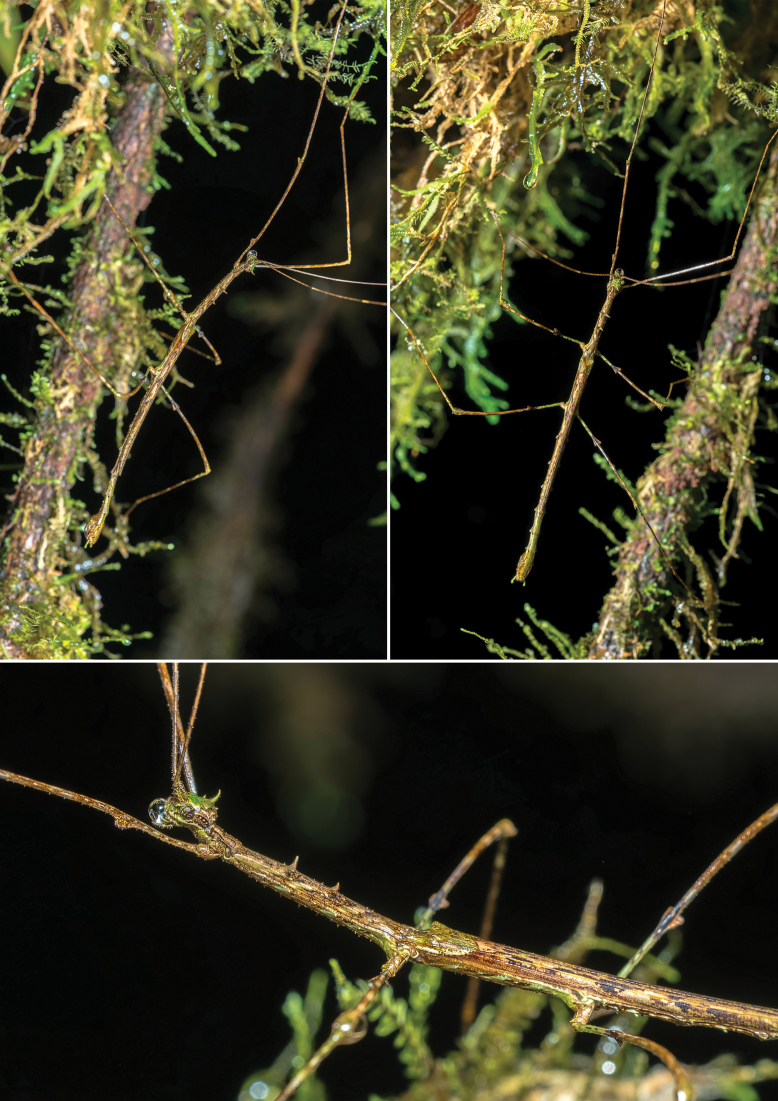
Males of *Trychopeplusmashpiensis* sp. nov. in their natural habitat.

♂♂ (Figs 4, 5, 7). Of medium size for the genus (body length 72.3–74.3 mm), has spines on the head and part of the body, although they are less numerous and smaller compared to those of the female. Similar to other known males of the genus, it has fully developed tegmina and alae.

The general colouration is similar to that of the female, though usually with less green. The tegmina and the costal area of the alae follow the same colouration pattern as the rest of the body, while the anal area of the wings is uniformly translucent ochre. The cerci are light green.

***Head*.** The head is very similar to that of the female, but with smaller ornamentation. However, the eyes are proportionally larger, with a diameter approximately 1.4 times the length of the genae. Ocelli absent. The antennae, although similar to those of the female, are proportionally longer, reaching and even slightly exceeding the total body length.

***Thorax*.** The thorax ornamentation is very similar to that of the female in terms of the number and position of spines, but these are much smaller, with the smallest considered as small protuberances rather than spines. The pronotum is approximately 0.75 times the length and width of the head, similar in shape to that of the female but with small protuberances instead of spines. The mesothorax is approximately 4.6 times longer than the pronotum and uniformly wide throughout its length. As in the female, the most prominent spines on the mesonotum are arranged in the first and second quarters of its total length; however, they are smaller than in the female, and the remaining spines appear as small protuberances. The metanotum is approximately one-third the length of the mesonotum and, unlike the female, does not have spines on the posterior margins. The tegmina are simple, with a slight elevation at the base of the wings to accommodate them; they are approximately 3.2 times longer than wide and cover a little more than one-third of the total length of the metathorax. The wings are approximately twice as long as they are wide and reach the abdominal segment VI.

***Abdomen*.** The abdomen is approximately the same length as the combined head and thorax (including the median segment). The median segment is 2.7 times longer than the metanotum. As in the female, segments II–IX have flattened lateral lobes near the posterior margin of each segment, but in the males, these are very small and uniformly sized across all segments. Abdominal segment II is approximately 0.5 times the length of the median segment and about three times longer than wide. Segments II–VII are all the same width, while VIII and IX are slightly wider. Segment III is the same length as segment II. From segment IV onwards, the length of each segment gradually decreases slightly. The median segment and tergites II–IX have a smooth surface without ornamentation.

Sternites II–VII have small blunt spines and a medial protuberance on the posterior margin of each segment. The anal segment is approximately 0.6 times the length of tergite IX and, as in the female, has a generally rounded outline, except for a small posterior medial incision and two triangular lateral projections near the base. The cerci are approximately 1.5 times longer than the anal segment, clearly extending beyond its posterior margin; they are slender and circular in cross-section. The vomer is shaped like a sharp spine and dorsoventrally flattened, with the basal half broadly widened. The poculum is shaped like an elongated scoop, with a small basal protuberance in the centre. In ventral view, it is asymmetrical, as the right outer margin presents a small expansion. The posterior margin is rounded and reaches halfway up the anal segment.

***Legs*.** The legs have similar morphology and ornamentation to those of the females, although they are longer, slender, and with less curved femora. All femora are longer than the combined head, pro-, and mesothorax. All tibiae are thin, more or less straight, slightly longer than the corresponding femur, and lack ornamentation. The basitarsi are slightly longer than the remaining tarsomeres combined and also lack ornamentation.

***Eggs*** (Fig. 2). Of medium size and atypical appearance for the genus (length 2.9–3.4 mm). The capsule is chestnut brown in colour, ovoid in shape, slightly higher than wide and longer than high. Its surface is adorned with numerous small crater-like pits arranged irregularly. There are no ornamental features such as hairs or fringes. The operculum rises prominently, forming a cone with a strongly rounded apex. The interior of the operculum is hollow and divided into three cavities by a thin, transparent membrane. Three openings in the walls of the operculum connect each of these cavities to the exterior, each surrounded by an irregularly elevated crown-like structure. A fourth opening, lacking an outer crown, is located at the vertex and seemingly provides access to the three cavities. The function of these structures remains unknown to the authors. The micropylar plate is outlined by a contour of reddish-brown colour. It resembles the silhouette of an avocado, approximately a little over half the length of the capsule. It is roughly twice as long as it is wide and is slightly shifted towards the polar area.

#### Measurements [mm].

Length including operculum 3.6–4.2, length 2.9–3.4, height 2.5–2.8, width 2.2–2.4, length of micropylar plate 1.9–2.1.

#### Distribution.

So far, the species is known only from the type locality, in the northwest of Pichincha Province, Ecuador.

#### Etymology.

The epithet *mashpiensis* refers to the Mashpi Reserve, where the species was discovered, in appreciation for the excellent treatment received from the lodge staff and the scientific team, as well as their significant conservation efforts.

##### ﻿Key to species of *Trychopeplus* Shelford, 1909

**Note.** The males of *T.spinosolobatus* and *T.thaumasius* remain undescribed.

♀♀ && ♂♂

**Table:** 

1	Prominent head and mesonotum ornamentation in the form of simple spines; mesofemora lobes simple with rounded margins	**2**
–	Prominent head and mesonotum ornamentation in the form of foliate lobes or branched spines; at least the medial pair of mesofemora lobes are compound and have irregular margins	**3**
2	Subgenital plate 1.6× longer than combined tergites VIII–X; femora with four pairs of rounded foliate lobes, one at the apex and the rest in the first, second, and third quarter of the corresponding femur length; Venezuela	***Trychopeplusspinosolobatus***
–	Subgenital plate 1.2× longer than combined tergites VIII–X; femora with two pairs of rounded foliate lobes, one at the apex and another in the first third of the corresponding femur length; NW Ecuador	***T.mashpiensis* sp. nov.**
3	Body length > 100 mm; pronotum without ornamentation; gonapophyses shorter than the subgenital plate; Ecuador, Colombia	***T.thaumasius***
–	Body length < 100 mm; pronotum with small spines; gonapophyses distinctly longer than the subgenital plate; Nicaragua, Costa Rica, and Panama	***T.laciniatus***

## ﻿Discussion

The inclusion of *Trychopeplusmashpiensis* sp. nov. within the genus *Trychopeplus* Shelford, 1909, is based on a careful evaluation of morphological and ecological criteria. Despite clear differences from the type species *Trychopepluslaciniatus* (Westwood, 1874), particularly in egg ornamentation, *T.mashpiensis* sp. nov. shares a suite of characteristics consistent with the typical morphology and ecology of the genus. According to Hennemann and Conle (2024), the distinctive morphological traits of *Trychopeplus* include large, numerous appendages and excrescences on the head and mesothorax, prominent lateral lobes on the abdominal tergites, as well as irregularly foliaceous lobes and teeth on the limbs.

Morphologically, *Trychopeplusspinosolobatus* (Redtenbacher, 1908) is the most similar species within the genus, as it exhibits appendages and excrescences on the head and thorax in the form of spines rather than foliaceous lobes, which distinguishes it from *T.laciniatus* and *T.thaumasius* Hebard, 1924. Unfortunately, only the female of *T.spinosolobatus* is known, leaving the morphology of its eggs uncertain, whether they resemble those of *T.laciniatus*, *T.mashpiensis* sp. nov., or are distinct to both of them.

One of the most significant findings of this study is the morphological differentiation between the eggs of *T.mashpiensis* sp. nov. and those of *T.laciniatus*, the only species in the genus whose eggs had been previously documented (Hebard 1922; Hennemann and Conle 2024). While the eggs of *T.laciniatus* are covered with long fringes (a feature previously suggested as a potential autapomorphy of the genus (Hennemann and Conle 2024)) the eggs of *T.mashpiensis* sp. nov. lack these fringes entirely (Fig. 2). This observation suggests a greater diversity in egg morphology within *Trychopeplus*, potentially indicating adaptive ecological diversification within the genus.

Hennemann and Conle (2024) hypothesised that the fringes on the eggs of *T.laciniatus* might aid in their attachment to moss or lichens, providing protection during embryonic development. However, this hypothesis is not applicable to *T.mashpiensis* sp. nov., given the absence of fringes on its eggs. Recent observations by one of the authors, during a short period of captivity (10 days), revealed that despite the presence of moss in the terrarium, females of *T.laciniatus* did not deposit their eggs on it, but instead threw them forcefully in random directions, a common behaviour among Diapheromerinae (Goldberg et al. 2015; Hennemann and Conle 2024). This egg-laying method makes it unlikely that the eggs would adhere to surrounding moss, especially considering that the fringes on the eggs of *T.laciniatus* remain retracted at the time of laying and for several hours afterwards (Hennemann and Conle 2024), preventing immediate adhesion. Büscher et al. (2024) examined the eggs of *T.laciniatus* using electron microscopy and observed that the egg hairs are, in fact, porous. Based on this finding, they suggested that these hairs might serve to absorb water, thereby preventing the egg from dehydration. Additionally, they proposed other hypotheses regarding the potential functions of these hairs, such as trapping air between them to prevent the embryo from drowning if submerged, or even playing a role in thermoregulation.

Despite the absence of long fringes on the eggs of *T.mashpiensis* sp. nov., this new species retains other characteristic features of the genus, such as the ovoid egg capsule, which is slightly longer than it is tall and wide; a pear-shaped micropyle plate displaced towards the polar area, sculpted similarly to the capsule; and an almost circular operculum with a prominently elevated outer margin, forming a hollow structure. The discovery of *T.mashpiensis* sp. nov.. highlights the need to reassess the characteristics used to define autapomorphies at the genus level, as the absence of fringes in the eggs of *T.mashpiensis* sp. nov. challenges the notion that this feature is diagnostic for the genus, as suggested by Hennemann and Conle (2024). Nevertheless, this intrageneric diversity is not entirely surprising, given the known interspecific variability observed in the eggs of related genera such as *Phanocles* Stål, 1875 and *Phanocloidea* Zompro, 2001 (Hennemann and Conle 2024).

**Figure 8.**
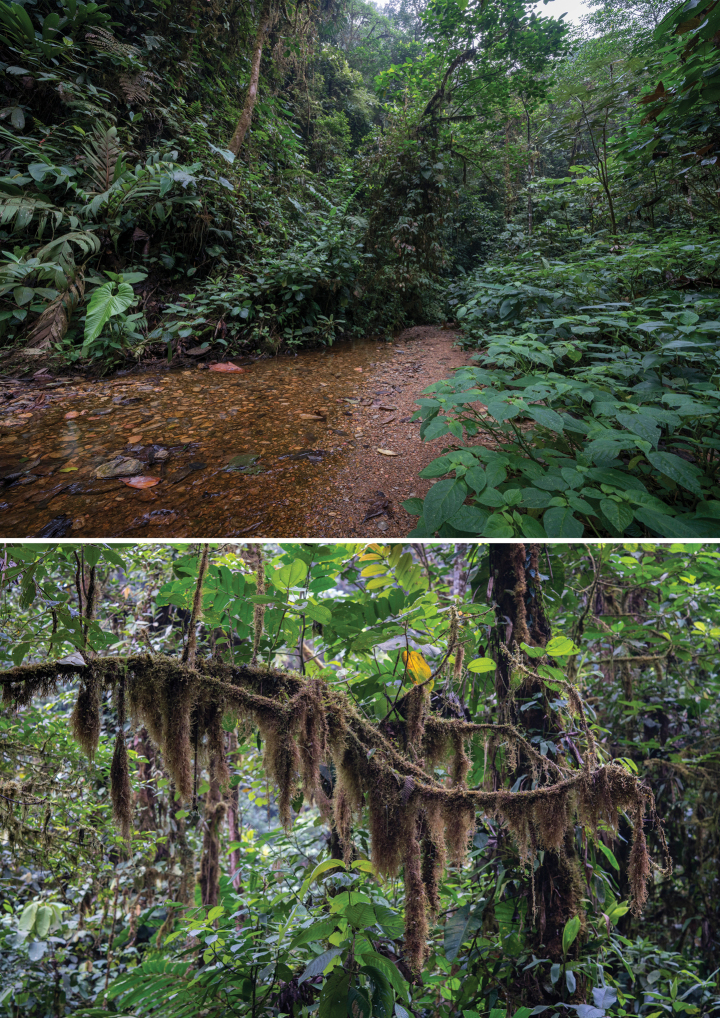
Natural habitat of *Trychopeplusmashpiensis* sp. nov. (Top), including an example of a microhabitat with concentrations of epiphytic mosses (Bottom) where they are commonly found resting.

The habitat, behaviour, and distribution of *T.mashpiensis* sp. nov. are consistent with those known for the genus (Fig. 8). All specimens, both nymphs and adults, were found at night in montane humid forests, at a height of 1.5 to 4 metres on trees with abundant epiphytic mosses. Similar habitats were observed by one of the authors during previous surveys, where *T.laciniatus* was found in Panama and *T.thaumasius* in Tungurahua Province, Ecuador.

In Monteverde, Costa Rica, *T.laciniatus* is known to feed exclusively on orchids such as *Prosthecheacampylostalix* (Rchb.f.) W.E.Higgins and *Oncidium* spp. (Orchidaceae; Kenji Nishida, pers. comm.). Additionally, one of the authors observed specimens of the same species feeding on *Maxillariaegertoniana* (Bateman ex Lindl.) Molinari in Chagres National Park, Panama. This is noteworthy, as there appear to be no other reports of Diapheromerinae feeding on orchids, suggesting this might be a unique characteristic of the genus. Nevertheless, although various types of epiphytic orchids were observed in the natural habitat of *T.mashpiensis* sp. nov., it was not possible to confirm whether any of them served as host plants, as no feeding specimens were observed during the surveys.

## ﻿Conclusions

The inclusion of *Trychopeplusmashpiensis* sp. nov. within the genus *Trychopeplus* Shelford, 1909 underscores the importance of taxonomic exploration and morphological revision in understanding Neotropical biodiversity. This study not only increases the number of known species within the genus to four, but also provides new insights into the morphological variability of eggs, which has significant implications for future identification and classification within the genus. The close phylogenetic relationship between *Trychopeplus* and *Phanocles* highlights the need for further research to clarify evolutionary relationships within the subfamily Diapheromerinae and to address the complexity of diversity within the genus.

## Supplementary Material

XML Treatment for Trychopeplus

XML Treatment for Trychopeplus mashpiensis

## ﻿Additional information

### Author ORCIDs

Oskar V. Conle https://orcid.org/0000-0001-5609-5107

Pablo Valero https://orcid.org/0000-0002-5471-3458

Frank H. Hennemann https://orcid.org/0000-0002-6547-4704

### Data availability

All of the data that support the findings of this study are available in the main text.
